# London's Ambulance Service

**Published:** 1909-04-03

**Authors:** 


### HOSPITAL ADMINISTRATION.

### CONSTRUCTION AND ECONOMICS.

### LONDON'S AMBULANCE SERVICE.

The report of the Departmental Committee appointed by the Home Secretary to inquire into the ambulance service in the metropolis, of which Sir Kenelm Edward Digby, K.C. (chairman), Lord Stamford, and Sir William Collins, M.P., were members, has issued its report. The scope of the inquiry was limited to "cases of accident and sudden illness occurring in streets and public places within the metropolis," but the Committee have taken evidence as to the means of conveyance to hospitals or elsewhere of persons lying in their homes or other private places, in urgent need of medical or surgical treatment.

The Committee arrive at the following conclusions:—

We think that it has been abundantly shown that the present system is gravely defective, and results in much preventable detriment and suffering by reason of the transport by unsuitable means of persons who have been injured or taken ill in the streets or other public places, and that the real evil arises from the great use which is made of ordinary vehicles such as cabs and vans even in serious cases which require the transport of the patient by the best means possible. We do not regard the wheeled litter as an ideal means of transport for "street cases," but it is highly serviceable and, apart from the question of speed in cases of the greatest urgency, fairly meets the requirements for safe, if not altogether comfortable, transport in most classes of cases. We regard a well-constructed and properly fitted rapid ambulance (horsed or motor) as a superior means of transport to the wheeled litter, and we should welcome, if practicable, the complete supersession of all other modes of conveyance by the rapid ambulance. Having regard, however, to considerations of expense, direct and indirect, we . . . consider that the wheeled litter service should be retained, subject to any improvements of the type of litter that may be possible, and, if necessary, additions to their number, and that the existing means of transport of "street cases" should be supplemented by the introduction and organisation of a sufficient number of rapid ambulances to deal with the more serious cases. We think that the introduction of rapid ambulances must in the first instance be tentative and experimental, but should be organised on lines which would enable the system to be extended so as to be capable of dealing with urgent cases other than "street cases."

The recommendations of the Committee, if adopted, will entail new legislation. They recommend:—

1. That the Metropolitan Asylums Board should be empowered by Act of Parliament to establish and maintain a non-infectious service of rapid ambulances available for dealing with the conveyance of persons suffering from accident or sudden illness occurring in streets or public places within the metropolis.

2. That for this purpose, and especially with the view to a complete separation of the non-infectious service of ambulances from the ambulances used for the conveyance of infectious cases, the Local Government Board should be empowered to frame regulations.

3. That the Metropolitan Asylums Board should, subject to such regulations, if any, as aforesaid, be empowered to enter into agreements with the Commissioner of Police, subject to the approval of the Secretary of State for the Home Department and Local Government Board, in respect of the following matters:—

(1) The number, position, size, and equipment of any new ambulance stations for the purpose of dealing with "street cases."

(2) The staff required for the service of the ambulances for the purpose aforesaid.

(3) The provision of the requisite signalling apparatus, and generally with reference to any matter necessary or incidental to the effective service of the ambulances.

(4) The use of the non-infectious ambulances of the Metropolitan Asylums Board for cases of accident or sudden illness occurring in the streets or public places within the Metropolitan Police District, but outside the County of London, on such terms as may be agreed on, including (if so agreed) a reasonable payment to the Metropolitan Asylums Board out of the Metropolitan Police Fund for the use of the ambulances for the purposes aforesaid and for any expenses connected therewith.

4. That the necessary powers should be given to acquire compulsorily any sites for stations or signalling apparatus required for the purpose of the ambulance service.

5. That the cost of and incidental to the provision and maintenance of the stations and their equipment, except in reference to any police officers who may be employed in or about such ambulance service, and except so far as regards the adjustment for those portions of the Metropolitan Police District which are outside the County of London, be borne by the Metropolitan Asylums Board, and no payment in respect of the use of the said ambulances shall be required from or on behalf of any person suffering from accident or sudden illness occurring in streets or public places.

6. That the Guardians of the Poor of any Union within the Metropolitan Police District be empowered, with the consent of the Local Government Board, to enter into an agreement with the Commissioner of Police, subject to approval as aforesaid, for the use of any rapid ambulance belonging to such Guardians for the conveyance of persons suffering from accident or sudden illness in streets or public places on such terms as may be agreed on, including, if so agreed, a reasonable payment from the Metropolitan Police Fund for the use of the ambulance.

Sir William Collins signs the report, subject to numerous reservations which he has embodied in a memorandum. We are generally in agreement with these reservations. We believe that they have a very momentous bearing upon the public issue, and that the fact that Sir William Collins was in a minority on the Committee is due to a want of apprehension and full knowledge of the needs and circumstances of the metropolis and of its local government, of which Sir William Collins is a master. We therefore hope that any legislation may follow the lines laid down by Sir William Collins rather than those embodied in the report of this Committee.

The whole subject is of the first importance. The report and memorandum are far too long to publish in full, and we advise our readers, especially all Londoners and members of Parliament, to study both carefully and in detail.

